# Assessment of pressure pain threshold at the cervical and lumbar spine region in the group of professionally active nurses: A cross‐sectional study

**DOI:** 10.1002/1348-9585.12108

**Published:** 2020-02-05

**Authors:** Anna Kołcz, Karolina Jenaszek

**Affiliations:** ^1^ Laboratory of Ergonomics and Biomedical Monitoring Wroclaw Medical University Wroclaw Poland; ^2^ Department of Physiotherapy Faculty of Health Sciences Wroclaw Medical University Wroclaw Poland; ^3^ Department of Neurological Rehabilitation Provincial Specialist Hospital Wroclaw Poland

**Keywords:** computerized pressure algometry, nursing practice, oswestry and neck disability index, pressure pain threshold, spinal pain syndromes, workplace ergonomy

## Abstract

**Objectives:**

The problem of spinal pain among nurses and lack of compliance with workplace ergonomy is increasing. The study aimed to assess the pressure pain threshold (PPT) at the cervical and lumbar spine in nursing staff.

**Methods:**

The sample of this prospective and observational study consisted of 30 female nurses with a mean age of 38.6 ± 11.1 years. The standardized Oswestry (ODI) and the Neck Disability Index (NDI) were used, as well as the Authors’ Designed Questionnaire (ADQ) was used to assess compliance with ergonomic principles. The PPT analysis using a computerized pressure algometer (CPA) was performed to examine the level of PPT.

**Results:**

A mild disability was found in 56% of nurses (NDI and ODI). A value of <4 kg/cm^2^ (CPA), indicating musculoskeletal overload was observed in 57% of subjects. Also, 60% of nurses work with a lying patient; 73.4% grabs the patient's armpits while transferring in bed; 16.7% never adjusts the height of the bed, and only 13.4% choose specialist footwear for work. There is a correlation between PPT values for trapezius and erector spinae muscles on the same side of the body in nurses with mild and moderate disability (*P* < .05).

**Conclusions:**

Pain complaints are associated with lower PPT of trapezius and erector spinae muscles and asymmetry of muscle tension. Also, it was noted that the lack of implementation of ergonomic principles by nursing staff affects their degree of disability.

## 1 INTRODUCTION

Nurses during their professional activity are exposed to the risk of work‐related musculoskeletal disorders, static and dynamic destabilization of the spine, resulting in chronic pain syndromes, thereby increasing the risk of injuries.^1^ Work‐related musculoskeletal disorders can be caused by inappropriate movement patterns during professional activity, a weak body posture causing deep spinal and abdominal muscles to malfunction, and inadequate implementation of ergonomic work practices.^2^

So far, many studies have pointed to unhealthy aspects of nurses' lifestyles.^3^, ^4^, ^5^ This is due to the specificity of a nurse's work in a specific ward,^6^, ^7^ work on shifts,^8^, ^9^, ^10^ improper dietary patterns and eating habits,^11^, ^12^ sedentary lifestyle and lower level of physical activity,^13^, ^14^ exposure to persistent occupational stress,^15^, ^16^, ^17^, ^18^ overwork and symptoms of burnout syndrome,^19^, ^20^ as well as frequent painful musculoskeletal complaints^21^, ^22^, ^23^ resulting from non‐compliance with the ergonomics guidelines.

It should be emphasized that in many hospitals, there are no ergonomic guidelines or even the wrong guidelines, there is limited or insufficient lifting equipment for nursing personnel. It is well known that manual lifting and other activities involving the repositioning of patients are associated with an increased risk of pain and injury to healthcare providers, particularly to the lower back. Exposition to external forces, performing the same motions in high frequency, and assuming body positions that are stressful to the movement system (reaching above shoulders, kneeling, squatting, leaning over a bed, or twisting the torso while lifting) are the most common risk factors.

Nurses in their everyday work often demonstrate non‐ergonomic behaviors. They repeatedly take on the wrong body position during their work activities, use incorrect grip, which is associated with the lack of proper conditions for the optimal performance of work activities. Due to a large number of patients in the wards, nurses often do not have free access to the patient's bed from different sides, as well as to the space around the patient's bed. Polish working conditions are often indicated as non‐ergonomic, which are confirmed in the previous studies. Juraszek et al,^24^ in their study among 205 respondents, found that 46.6% indicated limited access to bed as an obstacle to work. Juibari et al^25^ pointed out that the insufficient knowledge of nursing staff about workplace ergonomy in their profession is a significant problem.

In this aspect, two essential factors of postural control in a nurse's work are important, which significantly influence the musculoskeletal system overload in terms of biomechanical aspect. The first is a long‐term standing position that destabilizes the shoulder girdle and puts strain on the muscles of the cervical spine, leading to neck pain, movement restrictions, radiating pain in the upper limbs, or even dizziness and vision disorders.^26^, ^27^ The second stereotype is the position of excessive trunk hyperflexion, which is an extremely overloading position, especially during lifting and moving the patient, causing local lumbar and sacral pain or pain radiating to the lower limb.^28^, ^29^ Epidemiological studies show that the incidence of spinal pain among nurses ranges from 75% in the lumbar region to 60% in the cervical region of the spine.^30^ Squadroni and Barbini^31^ observed that 54% of nurses experienced multiple incidents of spinal pain during their professional careers; similarly, Karahan and Bayraktar^32^ reported that 87% of nurses experienced chronic or acute spinal pain syndrome.

In the era of evidence‐based medicine, increasingly precise and objective diagnostic and measurement tools are being developed, characterized by high repeatability of measurements or the possibility of integration with a computer system for data documentation.^33^ A computerized pressure algometer (CPA) is a more useful, objective, and precise measurement method in the diagnosis of soft tissue dysfunction than palpation testing. It is used to determine the individual pressure threshold of tissue sensitivity. The use of CPA allows measuring the pressure pain threshold (PPT) of soft tissues as the slightest pain‐inducing stimulus—defined by the International Association for the Study of Pain (IASP). According to Rosenberg and Sipko,^34^ a reduction in PPT indicates the possibility of overload symptoms of the musculoskeletal system. CPA is a reliable measurement device that supports the diagnosis of soft tissue trigger points and the myofascial pain syndrome.^35^

The primary outcome was to assess the PPT at the level of the cervical and lumbar spine in a group of professionally active nurses. The secondary outcome was to determine the degree of disability caused by existing spinal pain in the context of the implementation of the work‐related principles of ergonomy.

## 2 SUBJECTS AND METHODS

### 2.1 Design and settings

This prospective and observational study was conducted at the Department of Physiotherapy at the Wroclaw Medical University in Poland from September 2017 to December 2017. The office was adapted to the study by separating a place to take measurements, to fill in the questionnaire, and to rest before the study. The STROBE guidelines (Strengthening the Reporting of Observational Studies in Epidemiology) were followed.^36^

### 2.2 Qualification criteria

Inclusion criteria were as follows: (a) status of active nurse; (b) no diagnosis of chronic systemic diseases; (c) no current injuries to the movement system; and (3) voluntary written consent to participate in the study. In turn, the exclusion criteria comprised: (a) no professional activity as a nurse, (b) presence of systemic diseases and traumatic changes in the movement system, and (c) lack of consent to participate in the study.

### 2.3 Study participants

The study was carried out on a group of 30 active nurses with the mean age of 38.6 years (Min = 22, Max = 55, Me = 42.5, SD = 11.1). Mean body weight was 65.8 kg (Min = 49.6, Max = 100.1, Me = 63.6, SD = 10.5), mean body height was 165.4 cm (Min = 156, Max = 176, Me = 164, SD = 5.3), and mean BMI was 24 kg/m^2^ (Min = 17.8, Max = 35.9, Me = 23.5, SD = 3.6). The work seniority of 23.40% of the surveyed nurses was 0‐2 years, 13.30% was 3‐10 years, the next 13.30% was 11‐20 years, and 50% over 21 years.

### 2.4 Measurements tools

The test procedure consisted of four components. The study used two standardized and adequate tools in Polish language versions to evaluate pain‐related disability, that is, (a) Oswestry Disability Index (ODI) for the lumbar spine and (b) Neck Disability Index (NDI) for the cervical spine. Also, (c) Author Designed Questionnaire (ADQ) was used to evaluate the compliance of ergonomics principles during professional nursing activities. Also, an (d) objective PPT using CPA was performed to assess the individual pressure threshold of tissue sensitivity.

#### 2.4.1 Oswestry disability index (ODI)

The ODI questionnaire is composed of 10 parts that describe the ailments experienced in terms of pain intensity, lifting, sitting, sleeping, traveling, caring, walking, standing, social life, and changes in pain intensity. In each part, the examined person marked one of the six possible answers with a score of 0 to 5 points (a lower ODI score indicates lower pain).^37^

#### 2.4.2 Neck disability index (NDI)

The NDI questionnaire also consists of 10 parts: pain intensity, care, lifting, reading, headache, concentration, work, driving, sleeping, and rest. In each part, the examined nurse selected one of the six answers relating to her situation, receiving from 0 to 5 points (a lower NDI score indicates lower pain).^38^

#### 2.4.3 Computerized pressure algometer (CPA)

The CPA device model AlgoMed FPIX 50 (Medoc, Yishai, Israel) was used to measure PPT in the studied nurses. The examination was performed in a sitting position for the trapezius muscle. The measurement location was set by determining the point in the middle part of the transverse part of the muscle between the spinous process of C7 and the shoulder blades on both sides of the body. For the erector spinae muscle, the CPA test was performed in a forward lying position. The measurement location was set by determining the points on each side of the body, about 2 cm laterally from the spinous process of the L3. The points have been determined with particular accuracy, according to the manufacturer guidelines. The test was always performed by the same experienced and trained investigator.

The investigator applied an algometer head with a diameter of 1 cm^2^, perpendicularly at the designated points of the skin surface. When the pressure of the probe on the point within the examined muscles became painful, the tested person stopped the measurement using the button. The recorded compression values were saved by the algorithm in KPa and then converted to values in kg/cm^2^. Each point was measured three times at 10‐second intervals; then, the arithmetic mean was taken from all measurements of the point in one person.^39^ The pressure of CPA was used at a frequency of 1 kg per second for 4 seconds at every tender point up to 4 kg/cm^2^ pressure. According to the PPT value for healthy muscles adopted by the American College of Rheumatology (ACR), values less than 4 kg/cm^2^ indicates symptoms of musculoskeletal overloads resulting in pain.^40^, ^41^

#### 2.4.4 Author designed questionnaire (ADQ)

The ADQ has been developed for this study to determine the level of implementation workplace ergonomy principles by nurses. The ADQ consisted of eight questions to obtain general information on the positions taken during professional activities, the availability and use of patient handling equipment in the workplace, and the type of footwear used by the nurses.

### 2.5 Ethical considerations

The study was approved by the independent Bioethics Committee at the Wroclaw Medical University in Poland (no. KB–305/2018) and was conducted according to the Declaration of Helsinki and Good Clinical Practice guidelines. All participants gave their informed consent to participate in this study.

### 2.6 Statistical analysis

The results obtained in the study were analyzed statistically in Microsoft Office Excel 2007 and STATISTICA 10 (TIBICO Inc). Basic descriptive statistics were calculated: arithmetic means (M), standard deviation (SD), median (Me), minimum (Min), and maximum (Max). Qualitative variables are described in percentages and by pure numbers. Kruskal‐Wallis test and Spearman's rank correlation coefficient were used in the statistical analysis of the data. The Student's t test was used to analyze dependent and independent groups. The level of statistical significance was set at *P* < .05.

## 3 RESULTS

### 3.1 Disability level (ODI and NDI)

As many as 56% of the examined nurses showed a mild degree of disability resulting from pain (NDI and ODI), whereas moderate degree of disability was determined in 17% of respondents for cervical spine pain (NDI) and 14% for lumbar spine pain (ODI).

The analysis of the degree of disability in the NDI and ODI, respectively, indicates the lack of disability in 27% and 30% of people, mild disability in 56% according to both questionnaires and moderate disability in 17% and 14% of people.

The analysis of the results in the NDI and ODI questionnaires considering the number of nurses is shown in Table 1.

**Table 1:** Results of the NDI and ODI questionnaires according to the work seniority of nurses

Work seniority [y]	N	Min	Max	M	Me	SD
Total score in the NDI						
0‐2	7.0	0.0	17.0	5.4	4.0	6.1
3‐10	4.0	2.0	11.0	7.5	8.5	3.9
11‐20	4.0	3.0	10.0	5.5	4.5	3.1
>21	15.0	2.0	19.0	11.5	12.0	4.8
Total score in the ODI						
0‐2	7.0	1.0	14.0	5.4	4.0	4.5
3‐10	4.0	0.0	10.0	3.5	2.0	4.5
11‐20	4.0	8.0	17.0	12.3	12.0	3.7
>21	15.0	0.0	20.0	10.7	10.0	5.2

Abbreviations: M, mean; Max, maximum; Me, median; Min, minimum; N, number of participants; NDI, Neck Disability Index; ODI, Oswestry Disability Index; SD, standard deviation.

### 3.2 Pressure pain threshold (CPA)

It was found that most of the studied nurses had symptoms of musculoskeletal pain of overloading character. PPT values lower than 4 kg/cm^2^ in all four tested points were observed in 57% of respondents.

The distribution of PPT values divided into lower and higher than 4 kg/cm^2^ in all four tested points were as follows: within trapezius muscle 80% (n = 24) on the right and 73% (n = 22) on the left and erector spinae muscle 73% (n = 22) on the right and 63% (n = 19) on the left.

The PPT values divided into individual points for the tested muscles were as follows: for trapezius muscle an average of 3.3 kg/cm^2^ on the right and 3.1 kg/cm^2^ on the left and erector spinae muscle an average of 3.7 kg/cm^2^ on the right and 3.9 kg/cm^2^ on the left (Table 2).

**Table 2:** Results of PPT for trapezius and rector spinae muscles, including both sides

Tested muscle [side]	N	Min	Max	M	Me	SD
Trapezius muscle [R]	30	1.5	6.4	3.3	3.1	1.2
Trapezius muscle [L]	30	1.5	5.3	3.1	2.9	1.2
Erector spinae muscle [R]	30	1.4	8.2	3.7	3.2	1.7
Erector spinae muscle [L]	30	1.9	9.2	3.9	3.5	1.8

Abbreviations: L, left side; M, mean; Max, maximum; Me, median; Min, minimum; N, number of participants; PPT, pressure pain threshold; R, right side; SD, standard deviation.

The results of the analysis of PPT values for each muscle on both sides of the body are presented in Table 3.

**Table 3:** Results of PPT for trapezius and rector spinae muscles, including both sides, dividing values lower (overload) and higher (normal) than 4 kg/cm^2^

PPT [kg/cm^2^]	N	Min	Max	M	Me	SD
Trapezius muscle [R]						
<4	24	1.5	3.8	2.8	2.9	0.7
>4	6	4.2	6.4	5.4	5.3	0.8
Trapezius muscle [L]						
<4	22	1.5	3.6	2.5	2.5	0.7
>4	8	4.2	5.3	4.8	4.8	0.4
Erector spinae muscle [R]						
<4	22	1.4	3.9	2.8	2.9	0.7
>4	8	4.4	8.2	5.9	5.7	1.5
Erector spinae muscle [L]						
<4	19	1.9	4.0	2.8	2.9	0.7
>4	11	4.3	9.2	5.6	4.8	1.7

Abbreviations: M, mean; Max, maximum; Me, median; Min, minimum; N, number of participants; PPT, pressure pain threshold; SD, standard deviation.

### 3.3 Workplace ergonomics (ADQ)

As many as 60% of nurses surveyed when working with a patient in bedside areas incline their torso forward instead of bending their knees relieving the lumbar spine; 73.4% grasp the patient's armpits when moving around the bed; 13.3% “frequently” ask the patient to grasp the neck when lifting or to move the patient around the bed; while 33.3% admit to “rarely” asking the patient to do so.

When asked about the frequency of adjusting the height of the bed to one's height when working with a lying patient, 36.6% answered that they do it “always,” 30% frequently, 16.7% rarely, and 16.7% more rarely, and 16.7% more never. The presence of patient handling equipment at the workplace was declared by only 36.6% of the surveyed nurses. Verifying the type of footwear used by the nurses surveyed, 63.3% wear flaps; 23.3% clogs, while only 13.4% choose specialist medical footwear.

In the survey, the following activities were also considered necessary: feeding the patient 46.7%, collecting material for examination 43.3%, injecting 33.3%, measuring blood pressure 36.7%, and conducting an interview 66.7%.

### 3.4 Selected correlations and comparisons

Statistical analysis of Spearman's rank revealed a statistically significant correlation between PPT value within the trapezius muscle on the right and left the side of the body in the group with mild disability (NDI) (r = 0.71; *P* < .05). In the same group, there is also a statistically significant correlation between PPT values for erector spinae muscle on the right and left side (r = 0.78; *P* < .05). In the group of nurses with moderate disability (NDI), there is also a correlation between the PPT value within the erector spinae muscle on the right side of the body, relative to the left side (r = 0.73; *P* < .05). Among the subjects qualified for the group of mild and moderate disability (ODI), there is a correlation between the level of PPT for trapezius and erector spinae muscles on the same side of the body (r = 0.76; *P* < .05).

**Figure 1:**
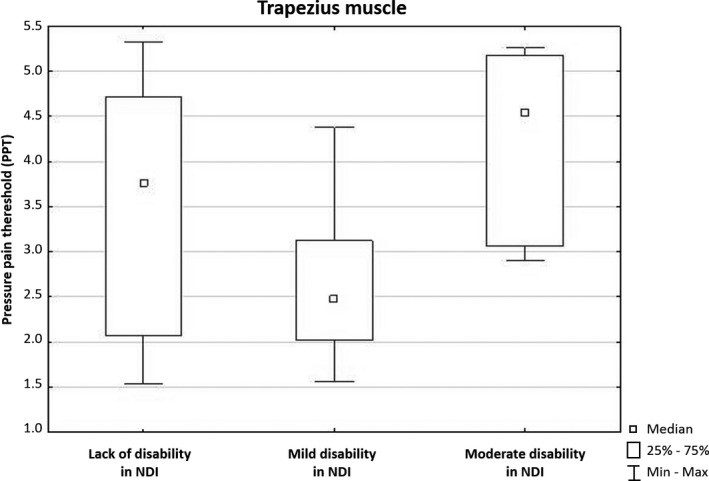
PPT results for trapezius muscle for both sides, according to different groups of the level of disability assessed with NDI. Max, maximum; Min, minimum; NDI, neck disability index; PPT, pressure pain threshold

The Kruskal‐Wallis test showed that there was a statistically significant difference in PPT of left trapezius muscle between mild and moderate disability (NDI) (*P* < .05). Among nurses with mild disability, PPT values were below 4 kg/cm^2^, whereas in the group with a moderate disability are between 3.0 and 5.25 kg/cm^2^ (Figure 1).

Comparing the level of disability (NDI and ODI) with the answers to the ADQ, it was observed that when working with a lying patient, non‐ergonomic posture (lifting without bending the knees) is assumed by 75% of nurses with moderate and 59% with mild disability.

All examined nurses with disability due to pain in both the cervical (NDI) and lumbar (ODI) regions have grasped the patient's armpits to move them within the bed. Among nurses with mild and cervical disability (NDI), 12% and 20%, respectively, admit that they often ask the patient to hold their neck when moving them in bed.

## 4 DISCUSSION

The incidence of spinal pain among nurses is a severe problem for those working in the healthcare sector. Dobrowolna and Hagner^42^ report that up to 90% of nurses may experience spinal pain. Similarly, study by Bilski and Sykutera^43^ report that about 73% of nursing personnel experience spinal pain more often than once a month. Tworek^44^ demonstrated that all of the nurses participated in the study suffer from spinal pain syndrome, of which 61.8% suffer everyday pain. The most common pain localization is lumbar spine (86.2%), and the majority of respondents determinate pain as moderate (60.2%).

In the study by Mikołajczyk et al^45^ in the group of nurses from the emergency department showed a statistically significant correlation between the degree of pain in the lumbar spine using visual analogue scale (VAS) and the degree of disability using Roland‐Morris Questionnaire (RMQ), and also showed a correlation between the degree of pain and the range of motion of the lumbar spine. Based on these studies, it can be concluded that increasing pain levels lead to reduced mobility in the lumbar spine, affecting the degree of disability and thus reducing the workability of nursing staff.

The ODI and NDI questionnaires were used in the present study to assess the influence of pain on the degree of nurses’ disability. Based on the results of the ODI, the nurses most often suffered from spinal pain, qualifying as a mild or moderate disability. In our study, none of the nurses showed any pain typical of severe disability; however, 27% of the respondents did not experience any pain in the cervical spine that would indicate disability.

Similarly, in studies by Maciuk et al^46^ and Pop et al^47^ in most studied nurses, the degree of disability was described as mild to moderate. When analyzing the results of the NDI, the majority of nurses were also characterized by a mild disability and to a lesser extent, by a moderate disability. Baumgart et al^48^ showed a similar quantitative distribution of results.

In our study, the CPA device was used to assess the occurrence of symptoms of musculoskeletal overload leading to pain in the cervical and lumbar spine among nurses. The PPT value (CPA), for healthy muscles, was assumed to be above 4 kg/cm^2^. The mean PPT value for trapezius muscle on the right and left the side of the body in the study group was 3.31 kg/cm^2^ and 3.13 kg/cm^2^, respectively; while for erector spinae muscle on the right and left side was 3.36 kg/cm^2^ and 3.85 kg/cm^2^, respectively. The study showed that in the majority of the studied nurses, PPT of the trapezius and erector spinae muscles was below 4 kg/cm^2^, which, according to the analyzed literature, indicates the overload of these muscles.

Rosenberg and Sipko,^34^ considering the impact of maintaining a normal sitting position on PPT and spinal pain; they concluded that PPT values are significantly lower in people with chest and lumbar spinal pain than in healthy people. Özdolap et al^49^ came to the same conclusion by testing PPT in people with chronic low back pain.

Based on the analysis of the results obtained, it can be concluded that among nurses with cervical and lumbar spine pain qualifying them for the group of mild or moderate disability, PPT values indicate the trapezius and erector spinae muscles hypertension and asymmetry of tension of individual muscle structures leading to overloading of the musculoskeletal system. In a case of muscles’ hypertension, the nociceptors localized in soft tissues send information to the central nervous system about abnormal tension in muscles or joints, which results in increased tension to secure the function of the motor system. Increased tension leads to pain, while the human body's defensive response to pain is to increase muscle tone, which leads to a pathological mechanism of the vicious circle of pain. The results of our study and the analysis of the results of other research indicate the need to implement specific principles of physioprophylaxis of spinal pain among nurses.

Nursing staff often have insufficient knowledge on workplace ergonomy, legal norms concerning lifting heavy objects or factors leading to the development of occupational diseases, as it was shown by Kowalczuk et al^50^ and Wyderka and Niedzielska.^51^ According to the literature of the last decade, the main factor leading to work‐related musculoskeletal overloads among active nurses is the lifting and carrying of patients. Maciuk et al,^46^ as the main reason for spinal pain among nurses, indicate the lifting of patients, as well as forced body positions taken during professional activities as well as frequent and dynamic changes of body position, performed in an accidental and non‐ergonomic manner. In the study by Tinubu et al,^52^ it was shown that working in the same positions for extended periods (55.1%), lifting or transferring patients (50.8%), and treating an excessive number of patients in one day (44.9%) were the most perceived work‐related risk factors of musculoskeletal irritations among nurses.

The results from ADQ in our study revealed that 60% of nurses do not bend their knees while working with a lying patient. Similar results were obtained by Maciuk et al^46^ and Juraszek et al^24^ Most of the women surveyed also do not bend their knees to relieve the lumbar spine during lifting and carrying. Our research has shown that the lack of knee bending and the performance of activities in a lying patient in an inclined position may affect the degree of disability. In the group of nurses with a moderate disability (ADI), 75% did not bend their knees while working with the patient.

To make their work easier, nurses can grasp the patient under their arms when moving the patient in bed or trying to verticalize the patient. This position forces an unfavorable body position with the simultaneous rotation of the spine and transfer of weight when the upper limbs are upright, which releases large shearing forces within the spine. The results show that 73% of nurses admit to using the patient's armpit grip when transferring or lifting the patient. Moreover, only 36.3% of the studied nurses always adjust the height of the bed to the activity level or body height of the patient, while 30% do it frequently and 16.7% rarely or never. This factor affects the degree of disability because our research has shown that all nurses with moderate disability catch the patient under the armpits during lifting and transferring.

Often observed behavior among nursing staff is also the patient's consent to the nurse's grip on the neck. Facilitating the verticalization or assurance of the patient may also directly contribute to the damage or overloading of the structures within the cervical region of the spine. Based on the results of our study, it was found that 20% of nurses in the moderate and 12 in mild disability allow the patient to hold their neck during transferring.

Dobrowolna and Hagner^42^ also draw attention to the biomechanical impact of lower limb pain on spinal pain in nurses. It was demonstrated that most nurses perform tasks such as feeding the patient, collecting test material, injecting and measuring blood pressure; they do so in standing position. Prolonged standing position contributes to problems with lower limb joints, which translates into a pain in both the lumbar and cervical spine.

It is noteworthy that Wyderka and Niedzielska^51^ pointed out that more than half of the nurses do not have access to medical equipment in their workplace. Lack of support equipment in hospital wards was reported by 52% of nurses surveyed by Kułagowska.^53^ In our research, 63.4% of nurses declared a lack of equipment facilitating patient transfer. Bilski and Sykutera^43^ showed a statistically significant correlation between the availability of facilitating equipment (eg, elevators, wheelchairs, electric beds) and a lower incidence of pain in the lumbar and sacral spine. Also, they reported that 44.13% of nurses did not have any knowledge about above‐mentioned facilitating devices and their usefulness at work.

Kwiecień‐Jaguś and Wujtewicz^54^ analyzing the workload of nursing staff working on anesthesiology and intensive care units, determined that there are symptoms of fatigue in terms of activity, physical symptoms, and motivation. A significant decrease in activity is manifested mainly by fatigue, sleepiness, fatigue of eyes and legs, dizziness, and lack of coordination. Fatigue, pain, and responsibility associated with the work of a nurse lead to a stronger feeling of stress, which, in turn, translates into increased muscle tension.^55^

Physical activity and physiotherapy play an important role in the elimination of spinal pain. The literature examining the painfulness of nursing staff was analyzed in terms of leisure time and pain management. The most common way to deal with pain is for nurses to use non‐steroidal anti‐inflammatory drugs. Based on the above activities and behaviors leading to spinal pain and musculoskeletal overloads, attention should be paid to increasing the nurses’ knowledge on the principles of ergonomics and the consequences of not complying with them.

It should be highlighted that our study revealed that nurses without disability and with moderate disability in NDI showed similar PPT values for trapezius muscle, while those with a mild disability had the lowest value than others. We assume that nurses with moderate disability present a higher levels subjective pain threshold in CPA due to the mechanisms of adaptation (habituation) to the persistent pain. This potential relationship may be the result of a specific adaptation to permanent pain for a prolonged period. Besides, it is worth considering the impact of a possible therapeutic effect after oral analgesic medications.

This study has a few potential methodological limitations that should be mentioned. First of all, the assessment of PPT using a CPA examination was performed at the level of the cervical and lumbar spine in a group of professionally active nurses. However, these measurements should be extended to different parts of the body, especially predisposed to work‐related musculoskeletal irritations and pain episodes. Myofascial overloads should be tested more globally, taking into account the axial pain, left‐ and right‐sided pain, and upper and lower segment pain or extremities pain. Also, our study focused only on work‐related disorders in musculoskeletal system resulted from poor adherence of nurses with workplace ergonomy principles. However, it should be emphasized that ergonomics should be seen in the broader context including work organizations and working systems; thus, this complex attitude should be considered in future studies.

## 5 CONCLUSIONS

Mild and moderate disability is associated with lower PPT values of the trapezius and erector spinae muscles, which reveals a noticeable dissymmetry of muscular tension. Lack of implementation of ergonomic principles by nurses affects their degree of disability. Taking non‐ergonomic body positions by nurses in their everyday work contributes to the reduction of PPT values of the trapezius and erector spinae muscles. It is worth implementing regular training on workplace ergonomics as well as methods of prevention and elimination of factors leading to work‐related musculoskeletal overloads among nursing staff.

## DISCLOSURE

*Approval of the research protocol:* The study was approved by the independent Bioethics Committee at the Wroclaw Medical University in Poland (no. KB‐305/2018). The study was conducted according to the Declaration of Helsinki and Good Clinical Practice guidelines. *Informed consent:* The study was completely anonymous, and each of the nurses surveyed gave voluntary and written informed consent to participate in the study. *Registry and the registration no. of the study/trial:* N/A. *Animal studies:* N/A. *Conflict of interest:* Authors declare no conflict of interests for this article.

## AUTHOR CONTRIBUTIONS

Study design: AK and KJ; Data collection: KJ; Data analysis: AK; Manuscript writing: AK and KJ; Revisions for important intellectual content: AK.
